# Impact of the structural integrity of the three-way junction of adenovirus VA_I_ RNA on PKR inhibition

**DOI:** 10.1371/journal.pone.0186849

**Published:** 2017-10-20

**Authors:** Edis Dzananovic, Astha, Grzegorz Chojnowski, Soumya Deo, Evan P. Booy, Pauline Padilla-Meier, Kevin McEleney, Janusz M. Bujnicki, Trushar R. Patel, Sean A. McKenna

**Affiliations:** 1 Department of Chemistry, University of Manitoba, Winnipeg, Manitoba, Canada; 2 Laboratory of Bioinformatics and Protein Engineering, International Institute of Molecular and Cell Biology in Warsaw, ul. Ks. Trojdena 4,Warsaw, Poland; 3 Manitoba Institute for Materials, University of Manitoba, Winnipeg, Manitoba, Canada; 4 Laboratory of Bioinformatics and Protein Engineering, Institute of Molecular Biology and Biotechnology, Faculty of Biology, Adam Mickiewicz University, ul. Umultowska 89, Poznan, Poland; 5 Alberta RNA Research and Training Institute, Department of Chemistry and Biochemistry, University of Lethbridge, Lethbridge, Alberta, Canada; 6 DiscoveryLab, Faculty of Medicine & Dentistry, University of Alberta, Edmonton, Canada; 7 Department of Microbiology, Immunology and Infectious Diseases, Cumming School of Medicine, University of Calgary, University Dr. NW Calgary, Alberta, Canada; 8 Department of Biochemistry and Medical Genetics, University of Manitoba, Winnipeg, Manitoba, Canada; Florida Atlantic University, UNITED STATES

## Abstract

Highly structured RNA derived from viral genomes is a key cellular indicator of viral infection. In response, cells produce the interferon inducible RNA-dependent protein kinase (PKR) that, when bound to viral dsRNA, phosphorylates eukaryotic initiation factor 2α and attenuates viral protein translation. Adenovirus can evade this line of defence through transcription of a non-coding RNA, VA_I_, an inhibitor of PKR. VA_I_ consists of three base-paired regions that meet at a three-way junction; an apical stem responsible for the interaction with PKR, a central stem required for inhibition, and a terminal stem. Recent studies have highlighted the potential importance of the tertiary structure of the three-way junction to PKR inhibition by enabling interaction between regions of the central and terminal stems. To further investigate the role of the three-way junction, we characterized the binding affinity and inhibitory potential of central stem mutants designed to introduce subtle alterations. These results were then correlated with small-angle X-ray scattering solution studies and computational tertiary structural models. Our results demonstrate that while mutations to the central stem have no observable effect on binding affinity to PKR, mutations that appear to disrupt the structure of the three-way junction prevent inhibition of PKR. Therefore, we propose that instead of simply sequestering PKR, a specific structural conformation of the PKR-VA_I_ complex may be required for inhibition.

## Introduction

RNA-dependent protein kinase (PKR) is a key interferon-stimulated enzyme involved in the innate immune response to viral infection. PKR is a Ser/Thr kinase that consists of tandem copies of a conserved double-stranded RNA binding motif (dsRBMs, residues 1–169) at the N-terminal domain, and a C-terminal kinase domain [1]. Upon viral infection and subsequent production of viral dsRNAs, PKR binds viral dsRNA, which enables self-association and a conformational change resulting in auto-phosphorylation on two threonine residues (Thr446 and Thr451) that overhang the enzyme’s active site [2]. Phosphorylated PKR in turn phosphorylates its target substrate eukaryotic initiation factor 2α (eIF2α) at Ser51, which slows the translation of viral proteins, thus helping the host cell’s response [3–5]. Phosphorylation on Thr446 and Thr451 leads to full activation of PKR and it promotes substrate recognition and phosphorylation [6, 7]. Typically, activation of PKR follows a bimolecular reaction mechanism [8, 9].

**Fig 1:**
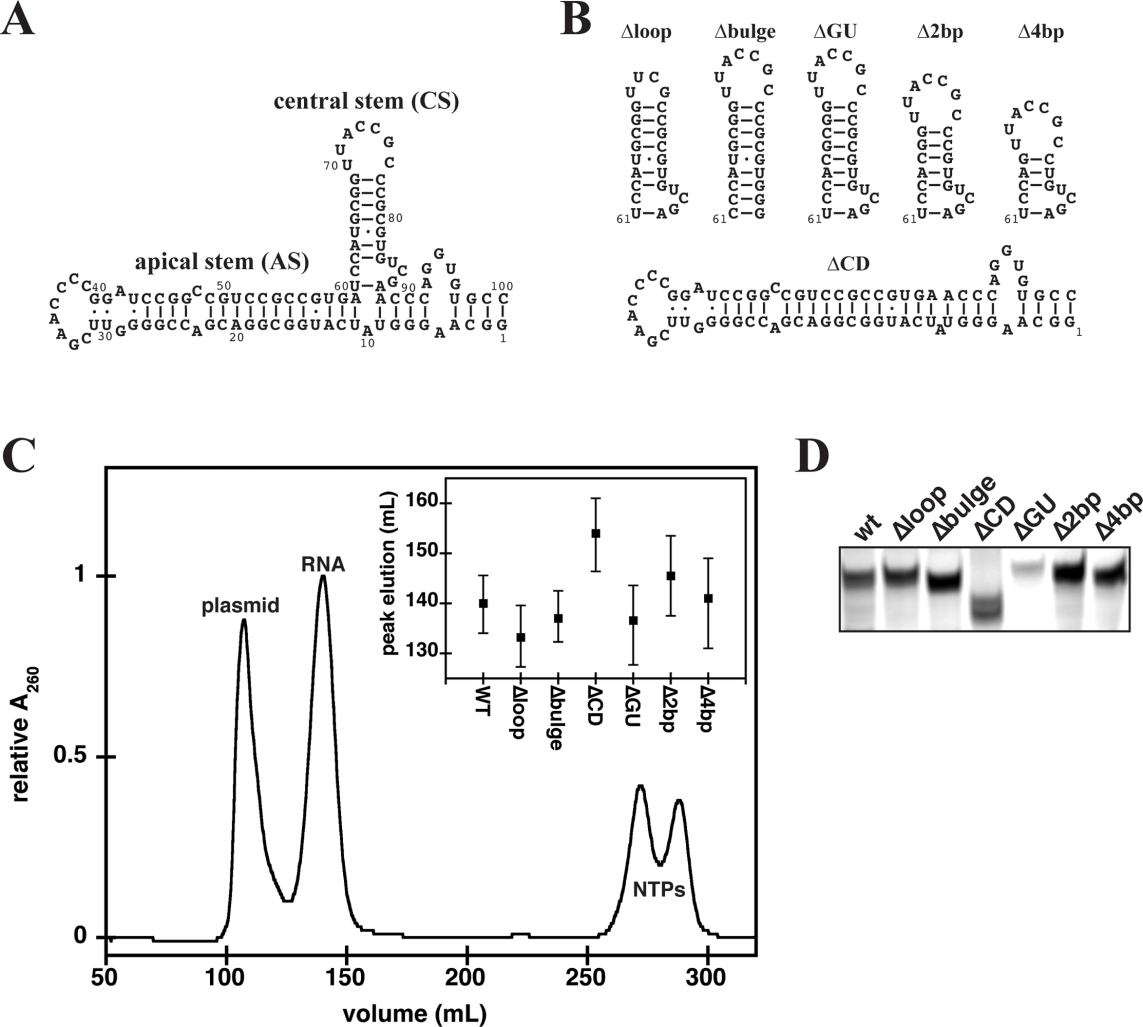
**(A)** Secondary structure of adenovirus VA_I_ΔTS (wt). **(B)** Schematic (not experimentally determined) representation of mutations in the CS of wt RNA and the ΔCD mutant that lacks the CS. **(C)** Purification of wt RNA by size exclusion chromatography (HiLoad 26/60 Superdex 75 column). Concentration of elution fractions was monitored by in-line spectrophotometric detection at 260 (solid line) and 280 nm simultaneously. The inset to the elution profile represents the elution range for the peak volume of each mutant RNA. **(D)** Native gel electrophoresis of wt RNA and its mutants. 2 μg of each RNA was loaded on 8% native TBE gel. Gels were stained with toluidine blue for total RNA.

To evade the host innate immune system viral countermeasures are used, including transcription of small non-coding RNAs that inhibit PKR via direct binding to the dsRBMs of PKR to prevent autophosphorylation [9, 10]. Adenovirus uses the host RNA polymerase III to transcribe virus associated RNA-I (VA_I_) that accumulates during the late stages of infection to inhibit PKR [11–14]. At the secondary structure level, VA_I_ consists of two stem-loops, apical (AS) and central (CS), and a terminal stem (TS) region that meet at a three-way junction (3wj) [15–18]. Functionally, the AS of VA_I_ is responsible for interaction with the dsRBMs of PKR, while the CS plays a pivotal role in the inhibition of PKR autophosphorylation [9, 10, 14, 19]. Most of the TS appears dispensable for PKR inhibition, as VA_I_ lacking 29 and 28 nucleotides from the 5' and 3' ends respectively (VA_I_ΔTS; Fig 1A) has no impact on affinity for or inhibition of PKR *in vitro* [10, 20]. Moreover, VA_I_ΔTS may represent a biologically relevant structure based on results demonstrated by the Dicer-processing of VA_I_ by the RNA interference machinery [20].

There are currently no high-resolution structures of either full-length PKR or VA_I_ or VA_I_ΔTS; however, high-resolution structures of N-terminal PKR_1-169_ [21] and C-terminal kinase domain [22] have been determined. The low-resolution structures of full-length PKR [23] and PKR_1-169_ alone or in complex with viral dsRNAs [24, 25] have also been reported using small angle X-ray scattering (SAXS). Together, both, low- and high-resolution data have given insight into the mechanism of activation/inhibition of PKR. NMR studies of PKR_1-169_ show that each dsRBM of PKR adopts a canonical fold required for dsRNA recognition, containing a 3-stranded antiparallel β-sheet flanked by two α-helices with the tandem dsRBMs joined by a 23 amino acid linker [21]. The C-terminal region of PKR encompasses a Ser/Thr kinase domain involved in PKR autophosphorylation and recognition and phosphorylation of target substrate. Structural studies on the kinase domain in complex with eIF2α detailed the overall Ser/Thr kinase fold including the Thr446 and Thr451 residues in activation loop overhanging the kinase active site that lead to PKR autophosphorylation and activation as well as the features required for target substrate interaction [22]. The dsRNA binding and kinase domains are joined by a flexible interdomain linker (80-residue). Previous studies have shown that significant flexibility in the two linkers (one between the dsRBMs, the other between the dsRBMs and kinase domains) exist, allowing for two distinct conformations of PKR; an extended “open” conformation where dsRBMs and kinase domains are not in contact, and a collapsed “closed” conformation [8, 23, 26–28].

The low-resolution structures of VA_I_ and VA_I_ΔTS reveal that the RNA adopts an extended shape in solution [25, 29]. Interestingly, tertiary structure computational models together with chemical probing suggest the potential presence of a pseudoknot in VA_I_ΔTS formed between the loop of central stem and a single-stranded region in the TS near the 3wj of the AS, CS, and TS [29]. The low-resolution structure of PKR_1-169_ in complex with VA_I_ΔTS also shows an extended shape in solution where nearly the entire length of VA_I_ΔTS tracks along both dsRBMs of PKR [. Computational models of VA_I_ΔTS-PKR_1-169_ complex derived using SAXS data for the complex, NMR structure of PKR_1-169_ and computational model of VA_I_ΔTS supports that both dsRBMs interact with VA_I_ΔTS and that pseudoknot formation via a specific 3wj is possible [25].

In this study, we have investigated the role of the specific structure of the 3wj in the mechanism of inhibition of PKR by the VA_I_ΔTS RNA. In order to establish the importance of the structure of the 3wj to PKR inhibition, we *in vitro* transcribed and purified VA_I_ΔTS RNAs with modest changes in the central stem of VA_I_ΔTS designed to impact formation of the 3wj while retaining full binding affinity for PKR via the AS. Electrophoretic mobility shift assays (EMSAs) were performed to confirm that the mutations do not affect binding affinity with PKR. *In vitro* autophosphorylation assays were then performed to determine the impact of mutation of the central stem on PKR activity. Results suggest that while alterations to VA_I_ΔTS can be tolerated, specific mutations to the central stem prevent inhibition of PKR. Dynamic light scattering (DLS), SAXS, and computational modeling tools were utilized to determine the impact of mutations on global tertiary structure and their inhibitory potential. Based on results from biochemical, biophysical and computational methods, we conclude that the structural integrity of the 3wj, potentially enabling pseudoknot formation, and not solely steric hindrance, is essential for PKR inhibition.

## Materials and methods

### In vitro transcription and purification of RNA

Sequences corresponding to the transcribed RNAs are shown in Fig 1. We refer to VA_I_ΔTS as a wild-type (wt) RNA for the remainder of the manuscript. Plasmid design for wt and mutant RNA was performed as previously described [30, 31]. DNA encoding the desired RNA sequence was cloned into pUC119 vector via HindIII/EcoRI sites. Plasmids were transformed into *E*. *coli* MAX Efficiency® DH5α™ Competent Cells (Life Technologies, Invitrogen, USA), purification effected by standard maxi-prep (Thermo Scientific, USA). Purified plasmids were linearized by BsaI restriction digestion in preparation for transcription. *In vitro* transcription using T7 RNA polymerase was performed as described previously [14, 31]. RNAs were purified using size exclusion chromatography (SEC) equipped with a HiLoad 26/60 Superdex 75 gel filtration column (2.6 x 60 cm, GE Healthcare Life Sciences, USA) in 50 mM Tris (pH 7.50), 100 mM NaCl, and 5 mM MgCl_2_ (Buffer 1) as reported previously [14, 31]. Purified RNAs were subsequently concentrated using appropriate spin concentrators (Millipore, USA). The purity of the transcribed RNA was verified by both denaturing and native polyacrylamide gel electrophoresis (in 1X Tris/Borate/EDTA (TBE) buffer). Twinned bands observed by native gel electrophoresis result from running buffer lacking cation present in Buffer 1; single bands are observed under denaturing conditions. RNA concentration was determined spectrophotometrically (NanoDrop2000c, Thermo Scientific), monitoring at 260 nm using the calculated extinction coefficients (ε_wt_ = 975000 cm^-1^M^-1^, ε_Δloop_ = 948500 cm^-1^M^-1^, ε_Δbulge_ = 963000 cm^-1^M^-1^, ε_ΔCD_ = 712000 cm^-1^M^-1^, ε_ΔGU_ = 975000 cm^-1^M^-1^, ε_Δ2bp_ = 937125 cm^-1^M^-1^, ε_Δ4bp_ = 902000 cm^-1^M^-1^).

### Protein expression and purification

Recombinant human PKR_1-169_ (N-terminal double-stranded RNA binding motifs with residues 1 to 169) as well as full-length PKR were expressed and purified as previously described [14]. Affinity purified proteins were subjected to SEC using a HiLoad 26/60 Superdex 75 gel filtration column (2.6 x 60 cm, GE Healthcare Life Sciences, USA) for PKR_1-169_, or HiLoad 26/60 Superdex 200 size exclusion column for full length PKR (all in 50 mM TRIS (pH 7.50), 100 mM NaCl, and 5 mM 2-mercaptoethanol (Buffer 2)). The elution fractions were monitored by means of absorbance at 280 nm and fractions containing purified protein were combined and concentrated using Millipore concentrator filters (Millipore, USA). Protein purity was confirmed by SDS-PAGE and concentration was determined using the known extinction coefficient as measured by UV-Vis spectrophotometry (NanoDrop2000c, Thermo Scientific, USA).

### Purification of RNA-protein complex

The wt-PKR_1-169_ and Δloop-PKR_1-169_ complexes were prepared by incubating purified protein in the presence of a 1.1-fold excess of RNA in Buffer 1 for 15 minutes at room temperature. After incubation, the mixture was applied on a HiLoad 26/60 Superdex 75 gel filtration column (2.6 x 60 cm, GE Healthcare Life Sciences, USA). The elution profile for both complexes showed two distinct peaks: a higher molecular weight peak corresponding to RNA-protein complex followed by a lower molecular weight peak corresponding to free RNA. Elution fractions for both complexes were assayed for the presence of RNA-protein complex via in-line spectrophotometer (at 260 and 280 nm simultaneously), and confirmed by native polyacrylamide gel electrophoresis. Fractions containing RNA-protein complex were pooled and concentrated using GE Healthcare Vivaspin 2 concentrators (GE Healthcare Life Sciences, USA). The purity of the complexes was assessed by the native polyacrylamide gel electrophoresis, whereas concentration was determined using the extinction coefficients of protein and RNA. The DLS profiles of wt-PKR_1-169_ and Δloop-PKR_1-169_ complexes showed no signs of aggregation across the concentration range at which the experiments were performed.

### Electrophoretic mobility shift assays (EMSAs)

EMSAs were performed by titrating RNA at 100 nM with increasing concentration (0–1000 nM) of PKR or PKR_1-169_ in 50 mM Tris, 100 mM NaCl (pH 7.0) and 5 mM MgCl_2_. The RNA and protein were mixed and incubated at room temperature for 10 minutes followed by addition of native load dye (0.02% bromophenol blue, 0.01% xylene cyanol FF, 10% glycerol in 1X TBE). The samples were loaded onto native TBE-PAGE gels and electrophoresis was performed at 80V and 4°C. The electrophoresis system (mini-protean 3 cell, Biorad) and the buffer (0.5X TBE) were kept on ice during for the length of the experiment. To visualize RNA-containing species, gels were stained with SybrGold (Invitrogen Inc., USA) for 5 minutes and imaged by the FluorChem Q System (Protein Simple, USA). Densitometry of free RNA bands was quantified using Alpha Imager Software provided with FluorChem Q System, and used to determine the fraction of bound RNA. *K*_*D*_ was determined by fitting the fraction of bound RNA against the protein concentration using a quadratic form of the binding equation as the amount of RNA used was not limiting [32].

$$fraction\;bound=\frac{[PKR]+[RNA]+K_{D}-\sqrt{{\left([PKR]+[RNA]+K_{D}\right)}^{2}-(4[PKR][RNA])}}{2[RNA]}$$

### Dynamic light scattering (DLS)

DLS data for RNA was collected prior to SAXS data collection to confirm that all the samples were highly pure and suitable for data collection. Samples were dialyzed for 2 hours at 4°C against Buffer 1 [50 mM Tris (pH 7.50), 100 mM NaCl, 5 mM MgCl_2_] prior to DLS analysis. After dialysis, samples were subjected to filtration through a 0.1μm filter (Millipore, USA) and equilibrated at 20°C. The hydrodynamic radius *r*_*H*_ and homogeneity (distribution of *r*_*H*_) of each sample was examined using the Zetasizer Nano S system (Malvern Instruments Ltd., Malvern, UK) equipped with a 4 mW laser (λ = 633 nm) as previously described [24, 25, 31]. DLS experiments for each sample were performed at multiple concentrations: 2.20–3.70 mg/mL for wt, 1.00–1.40 mg/mL for Δloop, 0.40–0.80 mg/mL for Δbulge, 1.50–3.10 mg/mL for ΔCD, 0.70–1.30 mg/mL for ΔGU, 1.80–2.80 mg/mL for Δ2bp, 0.40–0.80 mg/mL for Δ4bp, 1.10–1.70 mg/mL for wt-PKR_1-169_ complex, and 1.00–1.50 mg/mL for Δloop-PKR complex. DLS data were analyzed using the DTS software (Version 6.01, Malvern Instruments Ltd., Malvern, UK).

### PKR activation/inhibition assay

The *in vitro* PKR activation assay was carried out as follows. RNA samples at various concentrations (0–1000 nM) were pipetted into microfuge tubes on ice. Next a reaction mixture of 5X activation buffer (250 mM Tris at pH 7.5, 125 mM NaCl, 25 mM MgCl_2_, and 5 mM ATP), supplemented with PKR (100 nM) was added to tubes containing RNA to make a final buffer concentration of 1X. For the control reaction RNA samples were replaced by 0.0002 μg Poly I:C. The tubes were incubated at 30°C for 15 minutes and quenched by adding the 5X sodium dodecyl sulfate (SDS) load mixture to the reactions. 15 ng of protein was loaded on 10% SDS/polyacrylamide gel and transferred onto polyvinyl difluoride membrane to monitor PKR phosphorylation using anti-PKR phospho-Thr446 antibody (rabbit, ab47377, Abcam, with 1:2000 dilution) by means of western blot analysis as described previously [33]. In addition, anti-PKR antibody (mouse, ab32052, Abcam, with 1:5000 dilution) was also used for western blot analysis to monitor a loading control. Inhibition assays were carried out in a similar manner: 100 nM PKR was pre-incubated with the inhibitory RNAs ranging from 0–1500 nM for 10 minutes at room temperature. A mixture of 5X activation buffer and poly I:C was added to challenge the inhibitory RNAs and reactions were incubated at 30°C for 15 minutes and quenched by addition of 5X SDS load mixture. The western blot was developed as previously described and visualized using FluorChem Q System. Quantitation of PKR phosphorylation from at least triplicate experiments was performed using Alpha Imager Software. Band intensities were first corrected against the gel background and then against anti-PKR intensity (to ensure equal loading). Relative activity (on a scale of 0 to 1) was then determined relative to the poly I:C control.

### Small angle X-ray scattering (SAXS)

SAXS data for wt RNA were collected at 1.00, 1.90 and 3.10 mg/mL, 1.00, 3.00, and 4 mg/mL for Δloop; 0.40, 0.60, 0.80 mg/mL for Δbulge; 1.00, 1.50, 2.20 mg/mL for ΔCD; 1.30, 1.80, 2.30 mg/mL for ΔGU; 1.30, 1.70 mg/mL for Δ2bp and 1.40, 1.90, 2.40 mg/mL for Δ4bp. For the complexes data were collected at following concentrations- 1.30, 1.50, and 1.70 mg/mL for wt-PKR_1-169_ complex; and 1.25, 1.50 mg/mL for Δloop-PKR_1-169_ complex (all in Buffer 1). The data collection was performed at room temperature on a Rigaku 3-pinhole camera (S-MAX3000) equipped with a Rigaku MicroMax+002 microfocus sealed tube (Cu Kα radiation at 1.54 Å) and a Confocal Max-Flux (CMF) optics system operating at 40 W (Rigaku, USA). Scattering data were recorded using a 200 mm multiwire 2D detector. The data for all samples and buffer was collected for three hours for each sample within the range of 0.008 ≤ *s* ≤ 0.26 Å^–1^ and processed as previously described [34–36]. Briefly, the buffer scattering data was subtracted from the sample scattering data using the program PRIMUS [37] followed by merging of buffer subtracted data for all concentrations of each sample and the complex. The homogeneity of samples was assessed by Guinier analysis. The data were further processed using the GNOM program [38] to obtain radius of gyration (*r*_*G*_) and maximal particle dimension (*D*_*max*_*)*. The *ab initio* modeling for protein and individual RNA molecules was performed using the program DAMMIF [39]. The quality of the models was verified by the goodness of fit parameter (χ value) after each model calculation. *Ab initio* models for each sample and the complex were then rotated and averaged using the program DAMAVER [40]. Homogeneity of RNA sample was confirmed after SAXS experiments by native gel electrophoresis.

### Calculation of hydrodynamic parameters from ab initio models

The hydrodynamic properties were calculated for each model using the program HYDROPRO [41]. The atomic element radius of 2.9 Å was considered for HYDROPRO calculations according to the HYDROPRO manual. The density (1.004 g/mL) and viscosity (1.026 cPoise) of buffer was calculated using SEDNTERP [42] whereas the molecular weight and partial specific volumes for all RNAs were calculated using NucProt Calculator [43]. The partial specific volume of complexes were calculated using Equation 1 from [24].

### Computational modeling

Secondary structures were predicted for wt and mutant RNA mutants using ContextFold [44], a program that scored one of the most accurate single-sequence RNA structure predictions according to the CompaRNA benchmark [45]. In all cases but one, the effects of deletions on secondary structure were local, while in the Δ4bp variant, the deletion of the helix core has led to a rearrangement of the 3wj structure. Tertiary structures were predicted using RNA Masonry, a new method based on software developed in the Bujnicki laboratory, including the SimRNA method for RNA folding [46] and the library of recurrent 3D motifs available in RNA Bricks database [47]. Briefly, RNA Masonry uses the energy function established for the SimRNA method [46], and composes atomistic models by assembling existing fragments using a replica exchange Monte-Carlo algorithm, in which local structure is sampled by taking fragments from the database. The program exploits hierarchical organization of RNA structures, which are composed of regularly shaped double-stranded helices, and irregularly shaped loop motifs. It is therefore much faster than SimRNA, and generates full-atom models that agree with the input secondary structure. Here, we additionally restrained the fragment assembly process with goodness-of-fit to the experimental SAXS curve calculated using CRYSOL [48]. With this approach the number of degrees of freedom in an RNA model is reduced roughly to the number of junctions, which is particularly important owing to the limited number of unique observations available from SAXS experiment as well as the risk of over-fitting [49]. For building models with suspected pseudoknots, we have additionally used SimRNA to generate initial models with tertiary contacts between elements of secondary structure, and only then subjected these models to the refinement with RNA Masonry to improve the agreement with the experimental SAXS curve, while keeping the pseudo-knotted domain rigid.

The 3D models generated by RNA Masonry were regularized with the QRNAS program (Stasiewicz and Bujnicki, unpublished) that extends the AMBER force field with energy terms explicitly modeling hydrogen bonds geometry, base pair planarity and backbone conformation. It has been shown that QRNAS vastly reduces number of severe clashes in RNA models [50]. The final models were superposed onto the corresponding *ab initio* reconstructions using SUPCOMB [51].

## Results

### Purification of wt RNA and derivatives to probe three-way junction structure

To investigate the importance of the 3wj formed from the AS, CS, and TS on PKR inhibition, we *in vitro* transcribed wt RNA and six mutants in the CS that were predicted to retain the intact apical stem (AS) (Fig 1B). VA_I_ lacking the terminal stem was used as the wt RNA in this study as it is the minimal RNA able to inhibit PKR *in vitro* and is similar to the hypothesized Dicer-processed version of VA_I_ by the RNA interference machinery [10, 20]. The selected mutants probed the CS heptaloop by mutation to UUCG (Δloop), CS bulge at its base by conversion to perfect duplex (Δbulge), mid-CS GU base-pair by conversion to a canonical GC base-pair (ΔGU), CS length by stem shortening by two (Δ2bp) or 4bp (Δ4bp), or complete CS truncation (ΔCD). *In vitro* transcribed RNAs were purified using non-denaturing SEC in a buffer containing Mg^2+^ (Buffer 1; 50 mM Tris pH 7.5, 100 mM NaCl, 5 mM MgCl_2_) to separate template plasmid and small molecule contaminants from the desired RNA. wt RNA eluted as a single, compact peak (Fig 1C). The peak elution volume for all of the mutants is very similar to that of wt RNA, with the notable exception of ΔCD that lacks the CS and is of smaller size (inset to Fig 1C). Purified RNA samples were verified using native gel electrophoresis, where all RNAs share mobility similar to wt RNA, with the exception of the smaller ΔCD (Fig 1D).

**Fig 2:**
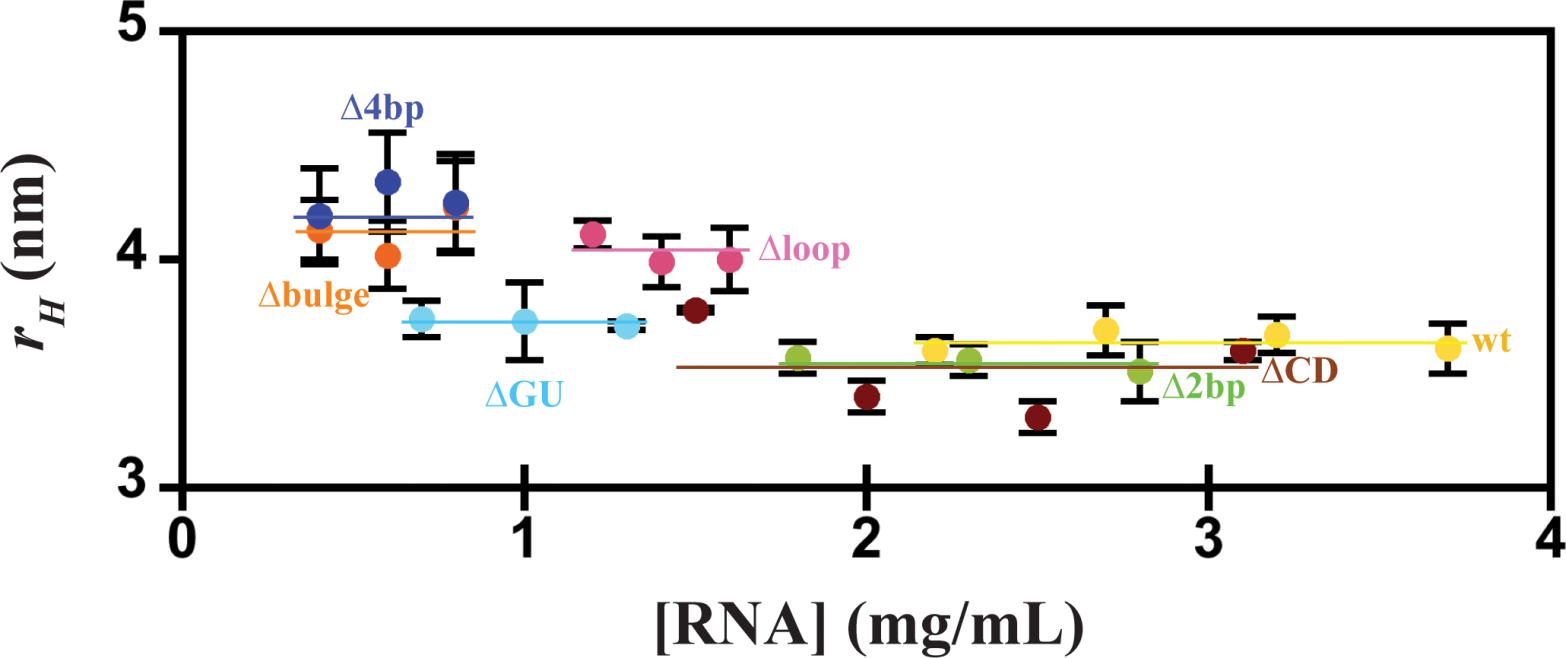
Dynamic light scattering of wt RNA and its mutants. The concentration dependence of the hydrodynamic radius (*r*_*H*_) was determined by DLS. The average (*r*_*H*_) value over the concentration range is highlighted with a horizontal bar. Error bars represent the standard deviation from at least 3 measurements.

Next, the homogeneity of each sample was studied using DLS to ensure that the RNA preparations did not display self-association over the range of concentrations ultimately used to study low-resolution structures by SAXS (Fig 2). Additionally, the average hydrodynamic radius (*r*_*H*_) for each sample at multiple concentrations was also determined (Table 1). Samples displayed no observable change in *r*_*H*_ consistent with the absence of self-association over the concentration range examined, suggesting that subsequent biochemical assay and SAXS studies were feasible. Reasonably comparable *r*_*H*_ values for wt RNA and mutants were observed, although Δ4bp, Δbulge, and Δloop demonstrated slightly larger values.

**Table 1:** Summary of hydrodynamic results.

RNA	Experimental						HYDROPRO^f^		
	*r*_*H*_ (nm)^a^	*r*_*G*_ (nm)^b^	*r*_*G*_ (nm)^c^	*D*_*max*_ (nm)^c^	χ^2^ value^d^	NSD^e^	*r*_*H*_ (nm)	*r*_*G*_ (nm)	*D*_*max*_ (nm)
**ΔCD**	3.52 ± 0.20	3.90 ± 0.20	3.70 ± 0.06	12.5	1	0.58 ± 0.03	3.40 ± 0.04	3.71 ± 0.04	12.6 ± 0.02
**Δ4bp**	4.26 ± 0.08	3.60 ± 0.30	3.63 ± 0.03	11.4	0.91	0.77 ± 0.04	3.68 ± 0.02	3.67 ± 0.03	11.5 ± 0.1
**Δ2bp**	3.54 ± 0.06	3.40 ± 0.10	3.60 ± 0.04	12.0	0.91	0.72 ± 0.03	3.55 ± 0.03	3.64 ± 0.04	12.1 ± 0.01
**ΔGU**	3.73 ± 0.01	3.7 ± 0.1	3.75 ± 0.04	11.8	1	0.80 ± 0.03	3.63 ± 0.03	3.78 ± 0.03	12.1 ± 0.01
**Δloop**	4.03 ± 0.07	4.3 ± 0.2	3.96 ± 0.05	12.4	1	0.95 ± 0.08	3.84 ± 0.03	4.00 ± 0.06	13.2 ± 0.12
**Δbulge**	4.13 ± 0.02	4.0 ± 0.2	3.87 ± 0.06	11.2	0.92	1.1 ± 0.06	3.84 ± 0.04	3.90 ± 0.05	11.7 ± 0.06
**wt RNA**	3.64 ± 0.04	4.1 ± 0.1	3.77 ± 0.05	12.5	1	0.78 ± 0.02	3.57 ± 0.04	3.78 ± 0.06	12.7 ± 0.02
**TSΔ21/PKR**_**1-169**_	3.90 ± 0.28	5.00 ±0.18	3.95 ± 0.07	13.2	0.9	0.67 ± 0.04	4.10 ± 0.02	3.97 ± 0.10	14.0 ± 0.02
**ΔLoop/PKR**_**1-169**_	5.30 ± 0.08	3.65 ± 0.15	4.27 ± 0.10	12.8	1	0.73 ± 0.05	4.55 ± 0.07	4.25 ± 0.10	13.3 ± 0.08

^a^, hydrodynamic radius determined using DLS in the presence of Mg^2+^

^b^, radius of gyration from Guinier analysis.

^c^, radius of gyration and maximal particle dimension determined using SAXS in the presence of Mg^2+^ using GNOM analysis.

^d^, parameter that compares *ab initio* model derived SAXS data with experimentally determined SAXS data

^e^, parameter that suggests agreement between multiple *ab initio* models

^f^, parameters calculated from *ab initio* models using program HYDROPRO

### Mutations in the central stem-loop of wt RNA do not significantly impair binding affinity to PKR

**Fig 3:**
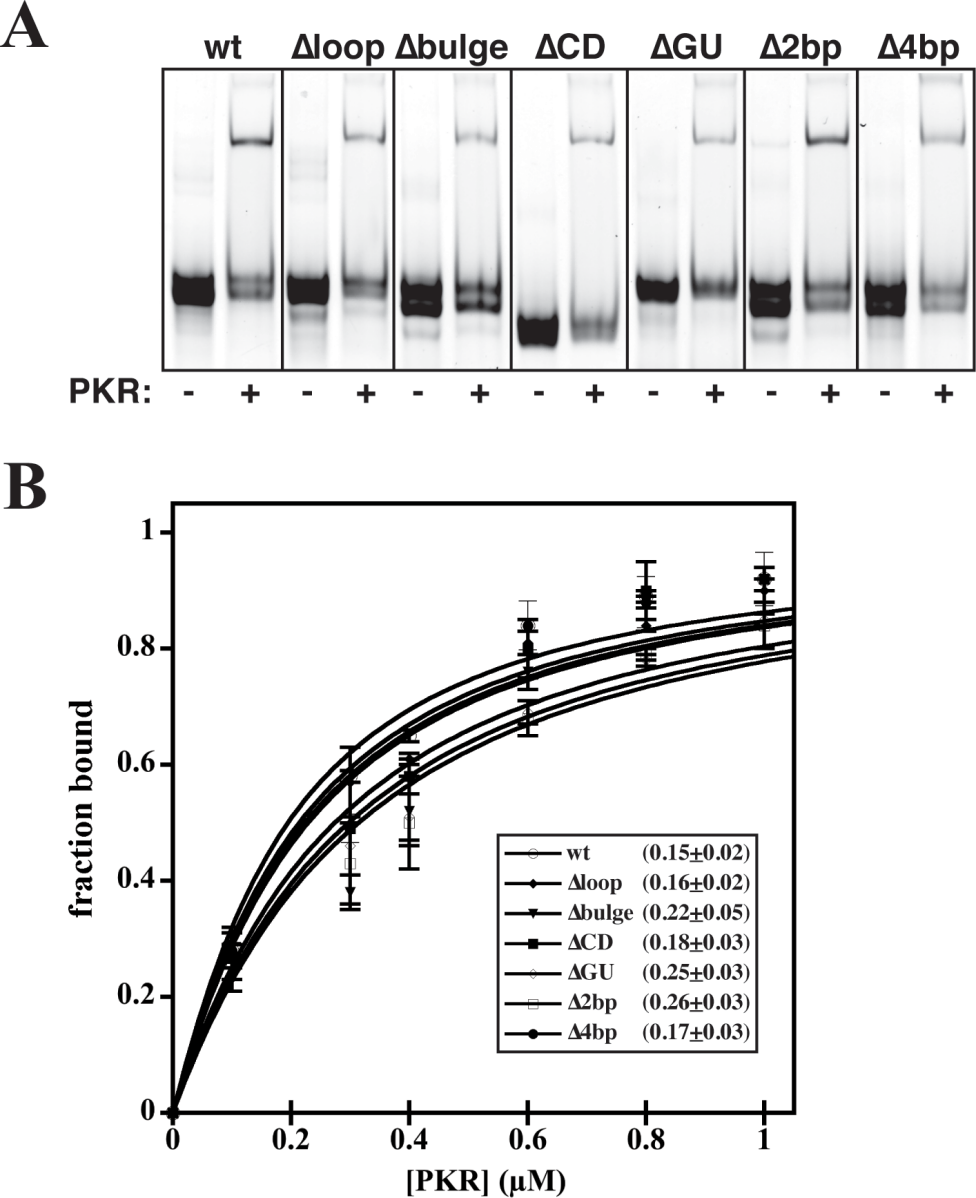
Central stem mutants display similar affinity to wt RNA. **(A)** EMSAs performed at a single concentration of RNA (100 nM) and protein (500 nM), stained with SybrGold dye for visualization. **(B)** EMSA quantification of RNA (100 nM) binding to PKR (0–1000 nM). Error bars represent the standard deviation from at least 3 measurements. Curve fitting and calculation of *K*_D_ was performed as described in Materials and Methods. The inset represents the determined *K*_D_ values (μM) derived from the curve fit.

Previous studies have shown that the CS does not make a high-affinity interaction with any region of PKR, and that the AS of VA_I_ is solely responsible for the interaction with the PKR [9, 10, 14, 19]. To confirm that introduced mutations of the CS did not impact binding affinity to PKR, EMSAs were performed at a single concentration of RNA (100 nM) and protein (500 nM) (Fig 3A). Under these conditions, both wt RNA and mutants result in detection of RNA-protein complex formation (using a fluorescent nucleic acid dye) that are of similar sizes near the top of the gel. To quantitatively compare affinities, RNAs were incubated with increasing concentrations of PKR and a gradual shift to a higher molecular weight species was observed by EMSA. As the shifted species stains far less intensely that the free RNA, quantitative comparison of affinity was achieved by determining the disappearance of free RNA to plot the fraction bound against the protein concentration (Fig 3B). Similar binding curves for wt and mutant RNAs were observed (much less than one order of magnitude in *K*_*D*_), implying that the mutations in the CS are not drastically impacting the AS structure nor the ability of RNAs to bind PKR.

### Specific mutations in the CS disrupt inhibition of PKR

**Fig 4:**
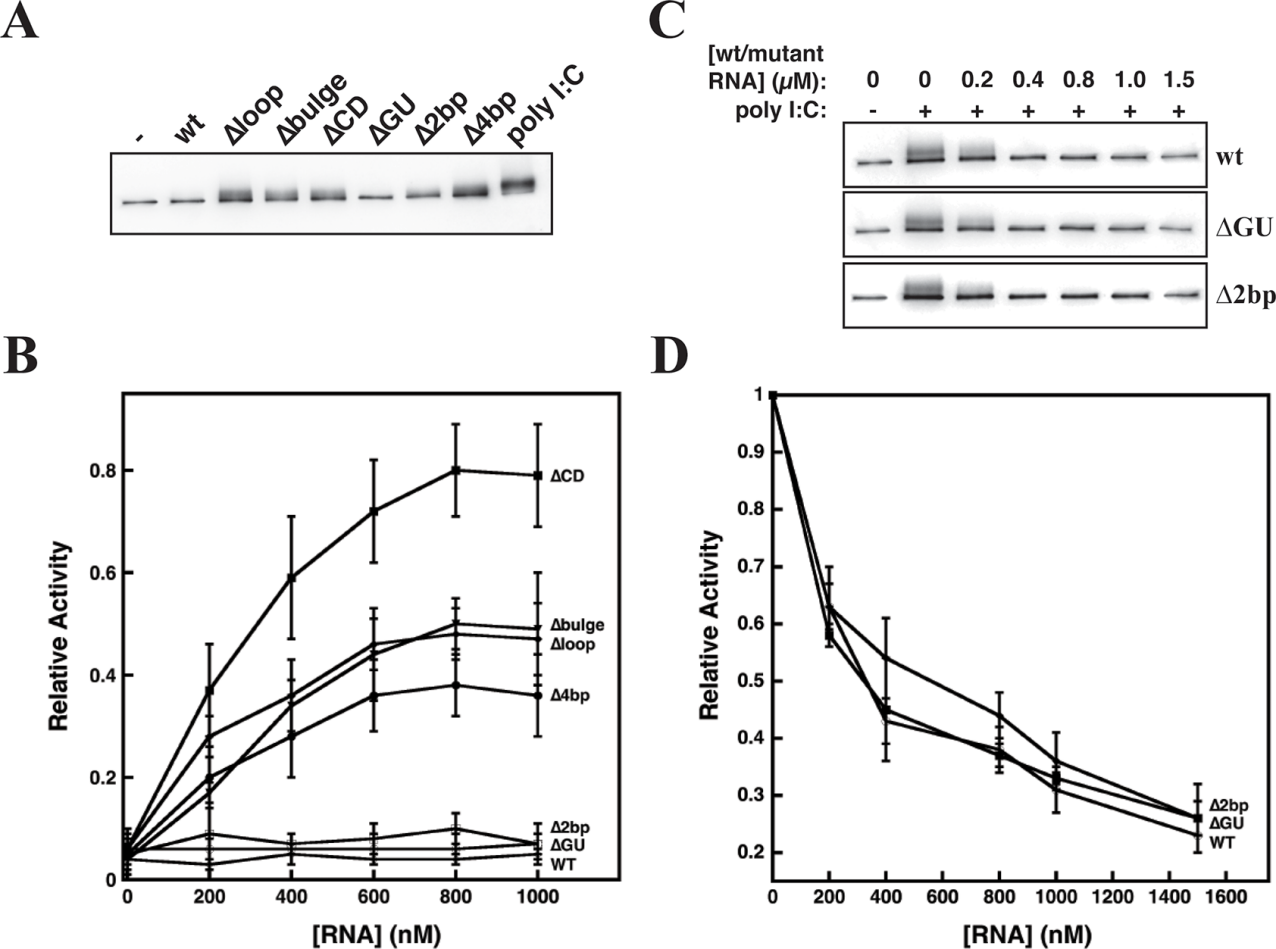
Biochemical assays to study the effect of mutations on PKR inhibition. **(A)** Activation of PKR in presence of wt RNA and CS mutants. PKR (100 nM) and RNAs (500 nM) were incubated for 15 min at 30°C. The phosphorylation levels of PKR were monitored by Anti-PKR Thr446 specific antibodies. **(B)** Quantification of PKR autophosphorylation in presence of variable concentrations of RNAs. Error bars represent the standard deviation from at least 3 measurements. **(C)** Inhibition of PKR by wt RNA, ΔGU, and Δ2bp. PKR (100 nM) and variable concentrations of RNAs were preincubated for 10 min at room temperature, followed by addition of 10 μg/mL poly I:C. The reaction mixture was incubated for further 15 min at 30°C. PKR autophosphorylation assays were performed, as described in the Materials and Methods. **(D)** Quantification of PKR autophosphorylation (100 nM) by poly I:C in the presence of potential inhibitors. Error bars represent the standard deviation from 3 measurements.

Recombinant human PKR activation loop autophosphorylation was assessed by incubation with wt RNA and mutants in the presence of ATP/Mg^2+^ with detection of Thr446 phosphorylation using immunoblotting (Fig 4A). In the absence of RNA (negative control), basal levels of PKR phosphorylation were observed. In the presence of synthetic dsRNA (poly I:C, positive control), an increase in antibody intensity and a slight upward migration on the gel are observed, both characteristic of PKR autophosphorylation. Consistent with its established role as a PKR inhibitor, wt RNA does not lead to PKR activation above negative control levels. Neither ΔGU nor Δ2bp lead to PKR activation. As expected ΔCD, which lacks the CS, acts as an activator of PKR. Interestingly, Δloop, Δbulge and Δ4bp also lead to PKR activation, demonstrating that these RNAs have lost their inhibitory activity (Fig 4A). We then repeated these experiments over a wide-range of RNA concentrations and with multiple replicates to semi-quantitatively compare the relative activity of these RNAs (Fig 4B). As expected ΔCD behaves as a potent activator of PKR, while Δbulge, Δloop and Δ4bp led to similar intermediate activation, well above the negative control. wt RNA, ΔGU, and Δ2bp were within error of the negative control.

To confirm that ΔGU and Δ2bp retained their ability to inhibit PKR (as opposed to not activating), increasing concentrations of these RNAs were incubated with PKR and then challenged with a potent dsRNA activator, poly I:C (Fig 4C and 4D). wt RNA, ΔGU, and Δ2bp are each potent inhibitors of PKR activation by poly I:C. Together the activation and inhibition assays strongly suggest that while all RNAs bind PKR with similar affinities, inhibition of PKR by wt RNA likely depends upon a specific structural conformation involving the CS.

### Specific central stem mutations affect the solution conformation

**Fig 5:**
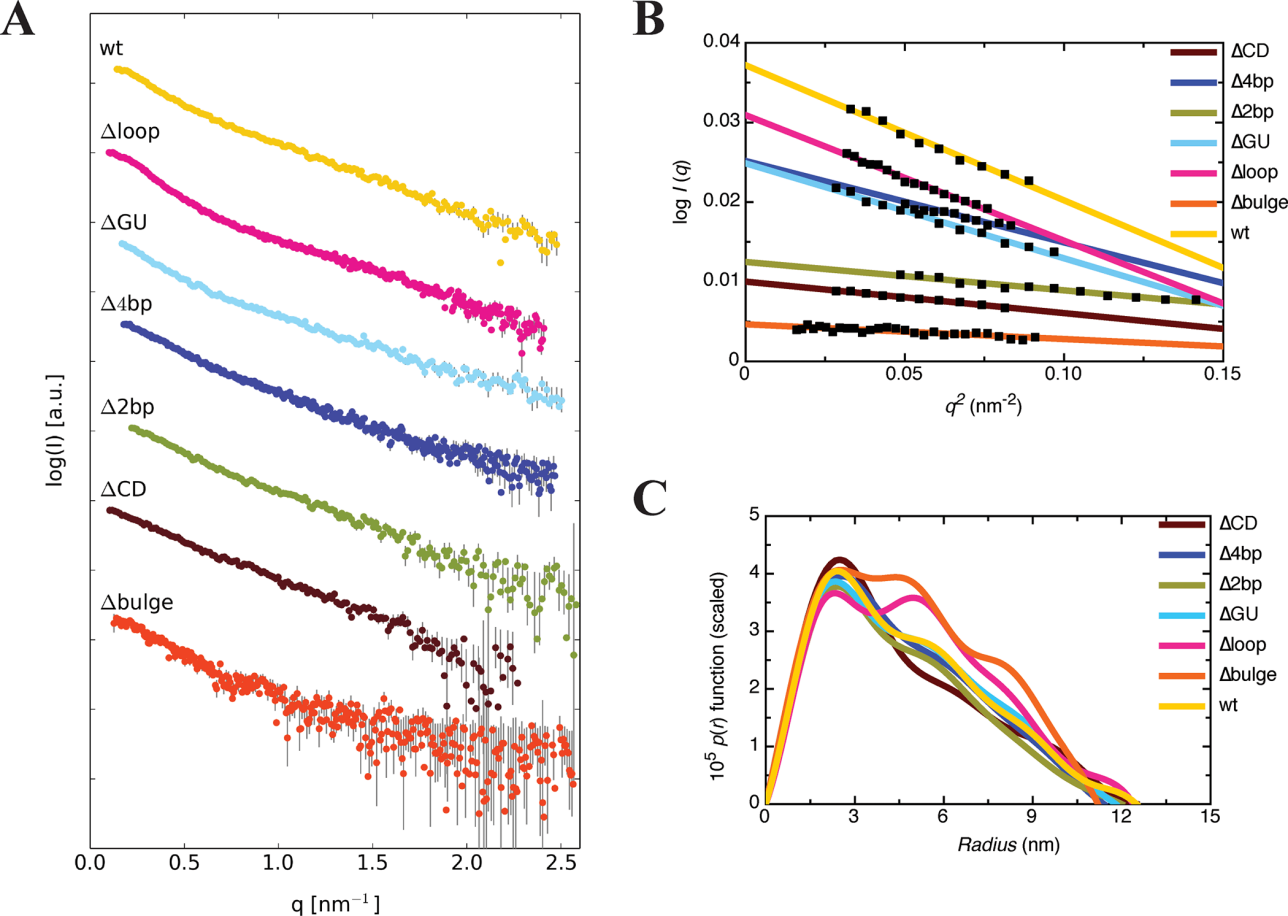
Characterization of wt RNA and CS mutants by SAXS. **(A)** SAXS scattering profiles, where each dataset represents the merged raw data from multiple sample concentrations. **(B)** A dotted line was fitted through the data at the low-*q* region of a plot of *log I*(*q*) *vs*. *q*^*2*^ (Guinier analysis) with an upper limit for *q*.*r*_*G*_ of 1.3 to obtain *r*_*G*_ from the slope for wt and mutant RNAs. **(C)** Dependence of the pair distribution function om the particle radius for each of the samples outlined in **(A)**.

To obtain structural insight on the impact of modest CS mutations on the conformation of the 3wj of wt RNA, we performed SAXS experiments. The scattering profiles of individual concentrations were merged to obtain a single scattering profile (Fig 5A). Primary analysis of the radius of gyration (*r*_*G*_) was performed using Guinier analysis, which also demonstrated the suitability of the data for further analysis (Fig 5B, Table 1). The merged scattering data was then used to generate the *p*(*r*) distribution function (distribution of electron pair distances in the sample) (Fig 5C). The *p*(*r*) distribution function of these RNAs adopt a skewed bell shape at short radii with an extended tail that is typical of an elongated molecule. For some mutants, most notably Δbulge and Δloop, the extended tails observed in the *p*(*r*) distribution adopt different shapes suggesting structural differences from wt RNA. To quantify these differences, the maximum particle dimension (*D*_*max*_) and *r*_*G*_ were determined from the *p*(*r*) distribution function. The *r*_*G*_ values determined for all RNAs obtained from GNOM analysis fell within a specific range (3.60–3.96 nm), with the largest values were observed for Δloop and Δbulge (Table 1). The *D*_*max*_ values determined by means of *p*(*r*) distribution were also relatively similar amongst the RNAs (11.2–12.5 nm), with Δbulge at the smaller end and wt/ΔCD at the larger (Table 1). Taken together, these results suggest that introduction of modest mutations, to the loop and bulge in particular, are impacting VA_I_ structure without its complete disruption.

**Fig 6:**
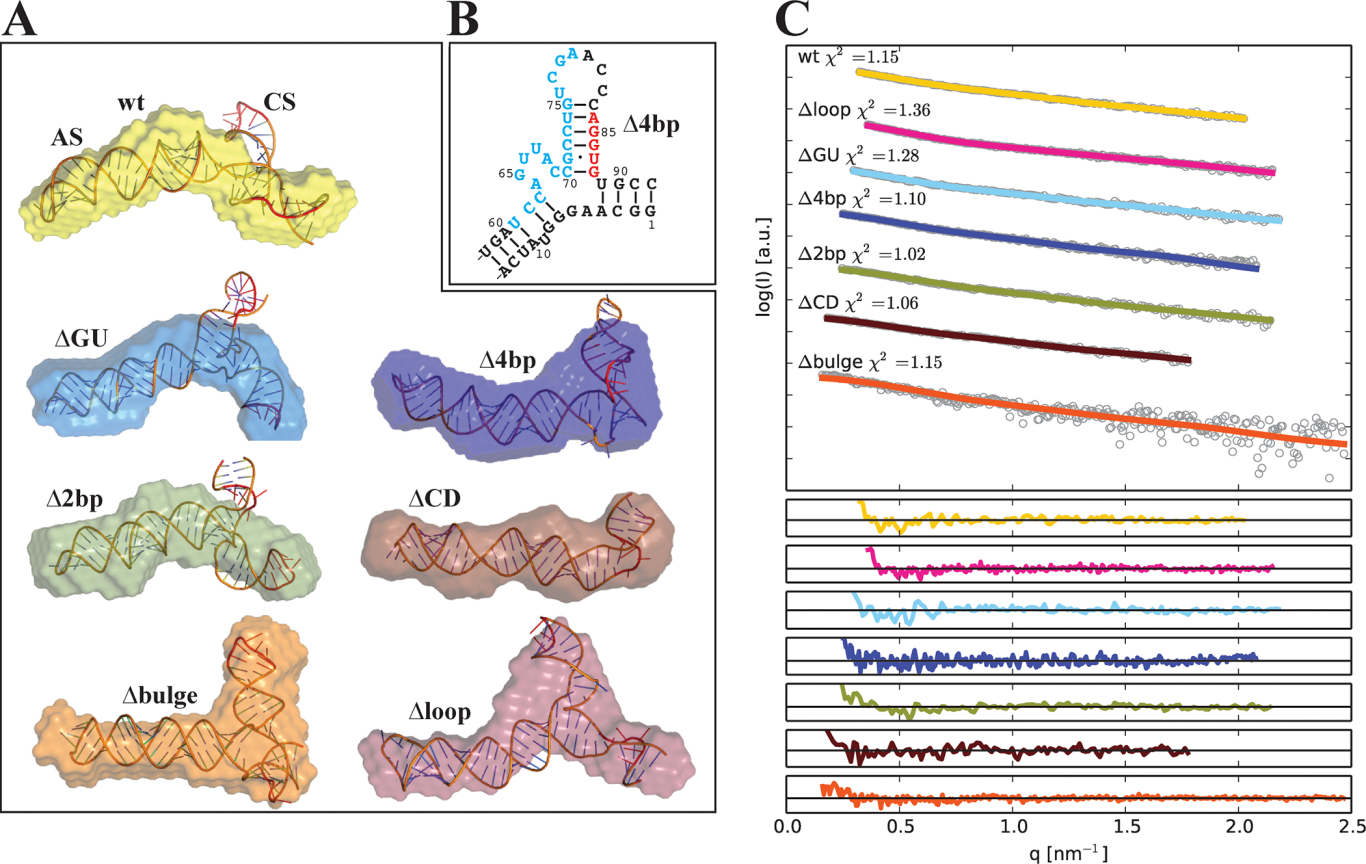
Solution conformation wt RNA and CS mutants. **(A)** Averaged *ab initio* model of wt RNA and CS mutants obtained from DAMMIF and atomic RNA models predicted using RNA Masonry. The models were aligned using SUPCOMB from ATSAS package. **(B)** Rearrangement observed in Δ4bp based on computational determination of the secondary structure. All other mutants demonstrated similar secondary structures to those shown in Fig 1B. **(C)** Fit between SAXS raw data (coloured lines) and computational model-derived SAXS data (black dotted lines), with residuals from the fit shown in the individual panels below.

To determine the average solution conformation of wt and mutants, the SAXS raw scattering data, *r*_*G*_, and *D*_*max*_ were used as constraints for generating *ab initio* models. Twenty models for each RNA were determined using the program DAMMIF [39]. The calculated χ^2^ value for individual models for each RNA was ~1 indicating good agreement between the experimental data and data calculated from *ab initio* models (Table 1). *Ab initio* models were then rotated, aligned, averaged and filtered using DAMAVER [40] that provided a normalized spatial discrepancy (NSD) of ~1 suggesting a strong agreement between the individual models. DLS experiments were carried out before (Fig 2) and after the SAXS experiments to ensure sample quality. An elongated solution conformation of wt RNA with a modest bend near the midpoint of the structure was observed, and is consistent with previously determined models [25] (Fig 6A, yellow). Noticeably, no obvious regions corresponding to the AS and CS are observed. ΔGU and Δ2bp present a similar overall shape as wt RNA, suggesting that these mutations have no discernable effect on VA_I_ conformation (Fig 6A, blue and green). ΔCD adopts a linear extended conformation consistent with an RNA that lacks the central stem and is almost completely A-RNA conformation along its length (Fig 6A, brown). Δ4bp adopts a conformation that is slightly less elongated and more globular at one end relative to wt RNA (Fig 6A, purple). Δloop and Δbulge show a large protuberance in the form of a distinct junction that is not observed in wt RNA (Fig 6A, crimson and orange). Hydrodynamic properties were calculated and compared with the experimentally determined properties, which resulted in a very good agreement between parameters from both approaches (Table 1), validating our *ab initio* models (in addition to the classical cross-checks of χ^2^ and NSD values).

### Computational structure determination suggests that the 3wj conformation is altered by specific mutations to the CS

To rationalize the determined solution conformation of the RNAs by SAXS, tertiary structure models were initially generated with the software RNA Masonry using only predicted secondary structure (without pseudoknots) and raw SAXS scattering data as restraints (Fig 6A) (see materials and methods for details). Predicted secondary structures correlated well with expectations (Fig 1B), with the exception of Δ4bp where the deletion causes local structural rearrangement, in which the position of the 3wj is shifted and an alternative helix is formed in place of the one destroyed by the deletion (Fig 6B). The final structure for each species was in good agreement with experimentally collected SAXS data (χ^2^ value in range of 1 to 1.36) estimated by means of CRYSOL (Fig 6C) [48]. ΔCD, as expected, presents a roughly canonical A-form RNA double helix that superimposes on the SAXS envelope well. Δbulge, Δloop and Δ4bp computational structures correlate very well with the low-resolution SAXS models, adopting a 3wj structure where the AS and CS are roughly perpendicular. While there are slight variations in the base-pairing pattern amongst the computationally predicted structures, the 3wj is still generally positioned in similar region in each RNA.

**Fig 7:**
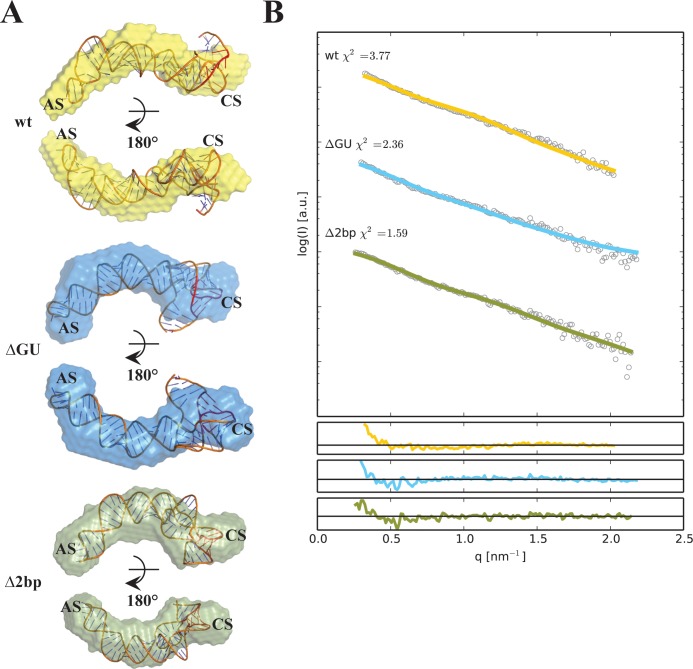
Computational structures of wt RNA, ΔGU and Δ2bp. **(A)** Atomic models were calculated using SimRNA3 and refined using RNA Masonry to improve fit to the experimental SAXS curves. Averaged *ab initio* reconstructions obtained from DAMMIF were superposed onto atomic models with SUPCOMB from ATSAS package. **(B)** Fit between SAXS raw data (coloured lines) and computational model-derived SAXS data (black dotted lines).

However, preliminary computational models of wt RNA, Δ2bp and ΔGU demonstrated significant deviation from the *ab initio* SAXS envelopes, which was intriguing given that these are the only RNAs that demonstrated inhibition of PKR (Fig 4). To determine whether an improved superimposition between the low-resolution SAXS envelope and the computational model could be achieved by introduction of a pseudoknot as a possibility, we recalculated high-resolution structures, allowing pseudoknot formation. First, models for wt, Δ2bp and ΔGU were built using SimRNA [46] (Fig 7A) to enable the possibility of the complex pseudoknot fold of the 3wj. Next, the geometry of AS in each of the models was refined with RNA Masonry to improve agreement with the experimental SAXS curve (Fig 7B). The results obtained using this approach revealed an extended conformation of wt RNA (χ^2^ value 3.77), where residues in the loop of the CS base-pair with a stretch near the 3'-end of the RNA allowing a pseudoknot conformation. Based on the computational models from Fig 7A, it is also evident that the ΔGU (χ^2^ value 2.36) and Δ2bp (χ^2^ value 1.59) display similar overall structures and are in good agreement with the low-resolution SAXS reconstructions. The imperfect fit to the SAXS curves is most likely a result of the vast reduction of the model’s degrees of freedom (conformations of the pseudoknotted domains were not altered to improve agreement with the experimental data). Any attempts to introduce the pseudoknot as an additional restraint for Δloop, Δbulge, Δ4bp resulted in obtaining even higher χ^2^ values and structures that did not fit well to the low-resolution SAXS reconstructions for these models. Together, these results suggest that inhibition may require a specific orientation of the 3wj to enable pseudoknot formation.

### Solution conformations of the dsRBMs of PKR in complex with wt and Δloop RNA

**Fig 8:**
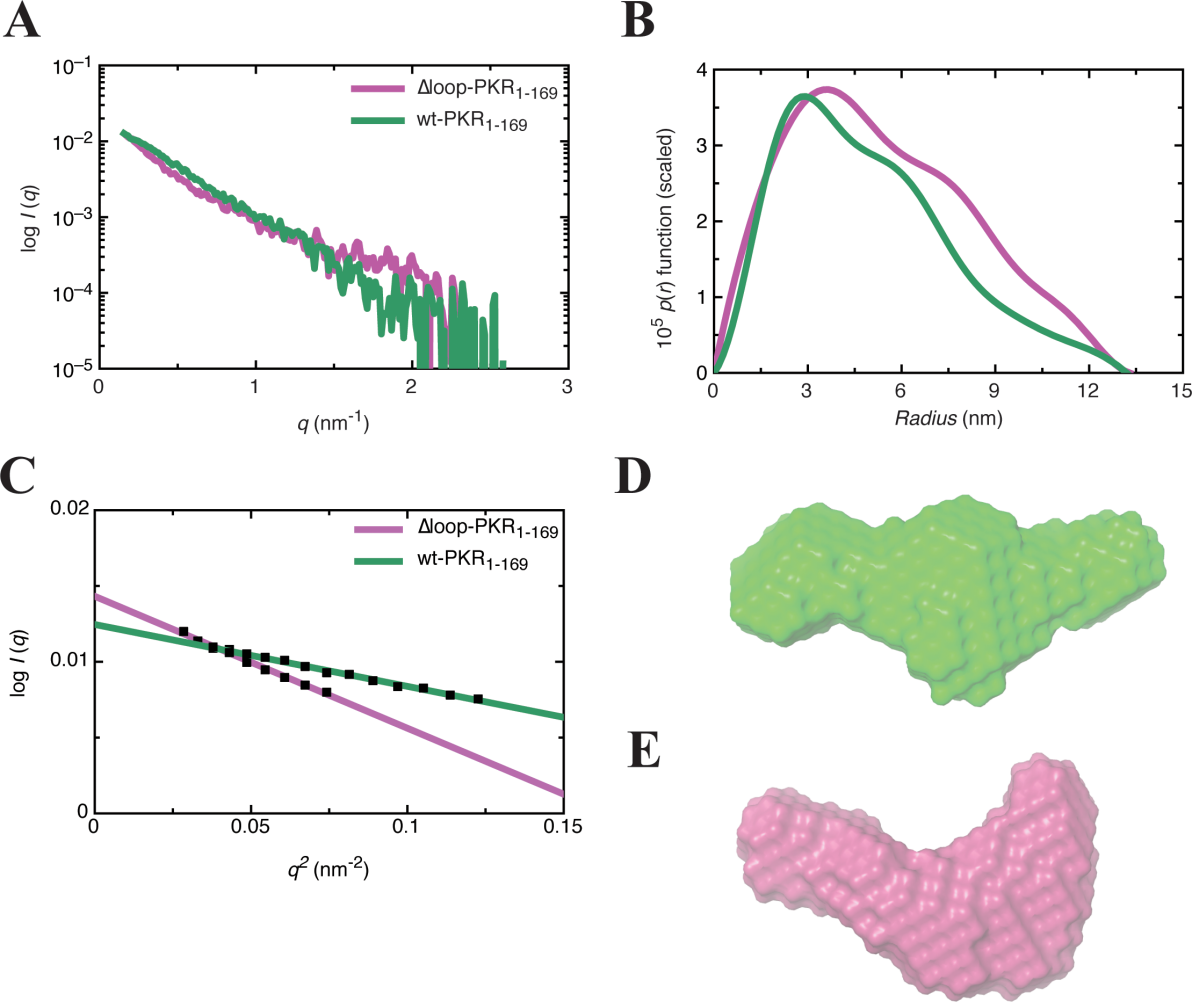
Characterization of complexes by SAXS. **(A)** SAXS scattering profiles for wt-PKR_1-169_, and Δloop-PKR_1-169_ complexes. Each data point represents the merged raw data from multiple sample concentrations. **(B)** The pair distribution function analysis of wt-PKR_1-169_, and Δloop-PKR_1-169_ complexes. **(C)** A dotted line was fitted through the data at the low-*q* region of a plot of *log I*(*q*) *vs*. *q*^*2*^ (Guinier analysis) with an upper limit for *q*.*r*_*G*_ of 1.3 to obtain *r*_*G*_ from the slope for both complexes. **(D)** A filtered model of wt-PKR_1-169_ complex. **(E)** A filtered model of Δloop-PKR_1-169_ complex obtained from *ab initio* calculations indicating overall shape of the complex in solution.

Previously, we reported the low-resolution structure of the wt-PKR_1-169_ complex in the absence of Mg^2+^ where PKR_1-169_ (dsRBMs alone) and wt RNA interact in a side-by-side orientation that involves entire length of PKR_1-169_ [24]. Recent reports suggest that the interaction between wt RNA and PKR is modulated by Mg^2+^ [29, 52]. We therefore extended our studies on wt-PKR_1-169_ complex in the presence of 5 mM MgCl_2_ (Buffer 1) to determine the effect of Mg^2+^ ions on overall structure and stability of the complex. The pair distribution function in the presence of Mg^2+^ is very similar to that previously determined in the absence of Mg^2+^ [25] (Fig 8A, 8B and 8C). The DAMAVER derived averaged model obtained from multiple *ab initio* models shows a one-to-one complex that is consistent with the previously observed solution conformation (Fig 8D). The calculated χ^2^ values show good agreement between the experimental and model-derived hydrodynamic parameters, and NSD values suggest that individual models are in good agreement with each other (Table 1).

We next determined the solution conformation of the Δloop-PKR_1-169_ complex (with Mg^2+^) as representative of an RNA mutant with a disrupted 3wj conformation (Fig 8A, 8B and 8C). While exhibiting similar *D*_*max*_ values, the pair distribution function of Δloop complex has distinct differences compared to that obtained for wt-PKR_1-169_, reflected in an *r*_*G*_ and *r*_*H*_ for Δloop-PKR_1-169_ being slightly larger than those of the wt-PKR_1-169_ complex (Fig 8E, Table 1). These differences, however, are consistent with those observed for the free RNAs (Fig 6). The *ab initio* model of Δloop-PKR_1-169_ complex generated by DAMAVER shows an extended structure in solution as suggested by the bell-shaped distribution function with a long tail (Fig 8B).

## Discussion

The antiviral properties of PKR are well established [3]. Viruses have evolved various strategies to circumvent host immunity. In the case of PKR, viruses produce small non-coding dsRNAs, including VA_I_, that interact with PKR and prevent its self-association, thereby inhibiting its antiviral effects. A recent review has highlighted the importance of VA_I_ as an essential pro-viral non-coding RNA [53]. The structural mechanism of inhibition of PKR by VA_I_ RNA is not fully understood, however previous *in vitro* studies have highlighted the importance of the CS of VA_I_ in the inhibition of PKR self-association [10–14, 19]. Recent studies have suggested that a pseudoknot forming between the CS and a single-stranded region (nucleotides 93–95, Fig 1) may play an important role in stabilizing the overall VA_I_ structure [29, 52]. Isothermal titration calorimetry, NMR and RNA footprinting studies previously suggested that the CS of VA_I_ does not make a direct high affinity interaction with any region of PKR [9, 10, 14, 19]. Therefore, while it is clear that the CS is required to sequester PKR in a 1:1 complex incapable of trans-autophosphorylation, it is unclear whether this is due to the CS imposing a steric block to PKR dimerization, or the CS being crucial to proper folding at the 3wj of the AS, CS, and TS so that inhibitory complex formation with PKR occurs. We therefore studied wt (VA_I_ΔTS) and CS mutants to improve our understanding of PKR inhibition. Binding affinity of these RNAs for PKR were not drastically different and consistent with previously published data [14, 54, 55]. Therefore, the structural and activity differences observed for RNA mutants cannot be accounted for by a simple increase or decrease in affinity.

Overall, the low-resolution structures of wt RNA and mutants in the presence of divalent cation support the formation of a pseudoknot requiring a specific structural 3wj orientation. This pseudoknot was originally proposed by Ma *et*. *al*. [56] and later confirmed by the Conn and Cole labs [29, 52]. Instead of observing the predicted perpendicular orientation of the CS and AS based on secondary structural information, wt RNA adopts an extended conformation in solution suggesting that the AS and CS form a more compact structure (Fig 6A). The Δloop and Δbulge mutants introduced a clear change in overall shape when compared to wt RNA (Fig 6A). We observe a clear conformational change in overall shape of Δloop, where a heptaloop is replaced with a UUCG tetraloop, suggesting that the loop sequence is important for the overall wt RNA fold despite its distal location from the 3wj. While converting the CS bulge to a perfectly double-stranded stem (Δbulge) was expected to introduce a more rigid conformation, a large protuberance in the 3wj region of this mutant was observed suggesting disruption of an important tertiary interaction. Interestingly, mutation of a GU base-pair to the more conformationally restricted GC bp in the CS had no such structural impact. Activation assays reinforce that mutations that disrupt the compact wt RNA fold behave as activators, and not inhibitors, of PKR (Fig 4). Complete truncation of the CS (ΔCD) shows substantially higher phosphorylation levels than wt RNA. Disruption of the compact pseudoknot structure with Δloop and Δbulge results in the loss of PKR inhibition despite increasing the potential for steric prevention of PKR autophosphorylation, arguing that a specific RNA conformation, as opposed to bulky steric features of VA_I_, is responsible for inhibition of PKR. CS stem length has also been shown to be crucial for PKR inhibition [10]. We demonstrate that while deletion of 2 bp in the CS has no large impact on structure or inhibition of PKR, the more aggressive deletion of 4 bp impacts the 3wj tertiary structure and its inhibition activity to a significant degree. We propose that these mutants are able to activate PKR instead of inhibiting PKR due to the loss of structural integrity of the 3wj.

In addition to the SAXS derived structures and activation/inhibition assays, the tertiary structures of wt and mutant RNAs based on computational approaches show that loss of function is likely related to the presence of a pseudoknot. Within the structural restraints of SAXS *ab initio* envelope, computational models predicting the pseudoknot structure (Fig 7A) between the central stem-loop and adjacent bulge are present within the mutants that retain the ability to inhibit PKR.

It has been reported that Mg^2+^ ions are required for tertiary stability of VA_I_, and that complex formation with PKR is enhanced in the presence of Mg^2+^ ions [29, 52]. Launer-Felty et al. [29] have previously proposed that both VA_I_ and VA_I_ΔTS (wt RNA in this study) may adopt a pseudoknot conformation in the presence of Mg^2+^, and our results support pseudoknot formation under similar conditions (albeit with a different overall predicted structure based on computational approaches). Based on our observations, the solution conformation of wt RNA in the presence of Mg^2+^ (Fig 6A, yellow) displays a more extended envelope with a correspondingly larger *D*_*max*_ relative to our previously determined SAXS solution structure of wt RNA in the absence of Mg^2+^ [25]. Comparison of the computational tertiary structures based on these SAXS results supports pseudoknot formation in the presence of Mg^2+^, whereas a perpendicular orientation of the CS and AS are observed in the absence of Mg^2+^.

Taken together, our results support the role that the formation of a specific structural conformation of the 3wj plays in inhibition of PKR. Overall the data presented here show that in addition to the previously proposed role of the central stem-loop of VA_I_ in PKR inhibition, the length of the central domain structure as well as its nucleotide composition are very important for the stability and the function of VA_I_ as an inhibitor of PKR. In summary, our results suggest the importance of studying such structural determinants in other non-coding RNA inhibitors of PKR. Studies are currently underway to test the importance of the 3wj in a cellular context.

## Abbreviations

3wj: three-way junction
AS: apical stem-loop
CS: central stem-loop
DLS: dynamic light scattering
*D_max_*: maximum particle dimension
dsRBMs: double-stranded RNA binding motifs
eIF2α: eukaryotic initiation factor 2α
EMSA: electrophoretic mobility shift assays
NSD: normalized spatial discrepancy
*p*(*r*) function: electron pair-distance distribution function
PKR: RNA-activated Protein Kinase
*r_G_*: radius of gyration
*r_H_*: hydrodynamic radius
SAXS: small angle X-ray scattering
SEC: size exclusion chromatography
TS: terminal stem-loop
VA_I_: adenovirus virus-associated RNA
VA_I_ΔTS (wt): VA_I_ lacking the terminal stem
VA_I_AS: apical stem-loop of VA_I_
